# Silicone Oil Migration to the Lateral Rectus Muscle: An Unusual Finding

**DOI:** 10.7759/cureus.40963

**Published:** 2023-06-26

**Authors:** Mohammed Elsheikh, Mubaraq Mustapha, Nicholas Bennett

**Affiliations:** 1 Ophthalmology, NHS Dumfries & Galloway, Dumfries, GBR

**Keywords:** retinal detachment surgery, silicone oils, intraocular silicone, lateral rectus recession, strabismus surgery

## Abstract

We report a case of silicone oil migration to the lateral rectus muscle discovered during otherwise uncomplicated strabismus surgery in a patient with multiple previous vitreoretinal surgical interventions. A 58-year-old female underwent strabismus surgery to correct a long-standing sensory exotropia. This patient underwent numerous previous vitreoretinal surgical interventions due to a complex history of retinal detachments with subsequent repairs, including inserting and removing intraocular silicone as an endotamponade. During this procedure, silicone oil cysts were discovered firmly adhered within the substance of the lateral rectus muscle. These cysts were subsequently removed either by rupture or whole excision, and specimens were sent for pathological examination. Microscopy confirmed sections of fat necrosis of the lateral rectus muscle with areas incorporating silicone oil. Following this, a lateral rectus recession and medial rectus resection were performed, and the exotropia was satisfactorily corrected. This report highlights an unusual complication of otherwise unchallenging strabismus surgery. Intraocular silicone oil introduced during vitreoretinal surgical procedures may present as cysts embedded on extraocular muscle, thus presenting a unique finding of strabismus surgery.

## Introduction

Silicone oil is frequently used as an internal tamponade during vitreoretinal surgery, such as the repair of retinal detachments. The extension of this substance to the extraocular region is a known complication of intraocular silicone oil use. Additionally, there have been increasing reports detailing the migration of intraocular silicone oil to the subconjunctival space [1-3], orbit [4,5], eyelid [6-8], and central nervous system [9]. This report describes a case in which multiple cysts were discovered embedded within the lateral rectus muscle in a female who underwent five vitreoretinal surgical interventions due to a complex history of retinal detachment and subsequent re-detachment.

## Case presentation

A 58-year-old female was referred by her general practitioner to our department on July 3, 2012, with reduced vision in her right eye of gradual onset. Initial examination revealed a best corrected visual acuity (BCVA) of 0.90 logMAR in the right eye and 0.50 logMAR in the left eye. Slit-lamp biomicroscopic examination revealed a significantly dense posterior subcapsular cataract (grade +++) in the right eye and a moderately dense nuclear cataract (grade ++) in the left eye.

Additionally, dilated fundus examination of the left eye revealed a chronic inferior retinal detachment as evidenced by a demarcation line. She subsequently underwent successful cataract surgery in both eyes, and consequently, BCVA had improved to 0.30 logMAR in the right eye and 0.06 logMAR in the left eye as of June 2013. As a result, our patient was successfully discharged from the hospital eye service.

On May 14, 2018, she was re-referred to us by her local optometrist due to a large superior macula-on retinal detachment with a U-shaped tear in the left eye. Visual acuity was noted to be 0.72, corrected to 0.12. She underwent a pars plana vitrectomy with cryotherapy and gas insertion on May 16. This was the first vitreoretinal intervention. A postoperative follow-up appointment one month later noted vision to be 0.60 logMAR in the affected eye. Unfortunately, she re-presented six weeks later with a re-detachment of her left retina and promptly underwent further retinal detachment repair on July 2, 2018, however, with silicone oil rather than gas insertion. By August 2018, the superior retina of the left eye was noted to be successfully re-attached. However, the inferior retina was found to be persistently detached, as previously noted. Hence, plans were formed to remove and replace the intraocular silicone oil with heavier silicone oil (Densiron 68).

By November 2018, both the superior and inferior retina of the left eye were noted to be attached. Thus, the intraocular silicone oil was removed 12 weeks later. Clinical examination following this intervention noted significant left-sided hypotony refractory to medical treatment, leading to the prompt re-insertion of silicone oil. Four weeks after this intervention, the intraocular pressure was adequately corrected; however, the BCVA in the left eye was only 1.00 logMAR. Due to this, she began developing sensory exotropia in the affected eye. Moreover, she also developed elevated intraocular pressure in her left eye secondary to long-term intraocular silicone oil. The intraocular pressure was satisfactorily controlled with bimatoprost/timolol combination eye drops. She was listed for strabismus surgery under a general anesthetic in April 2021.

**Figure 1:**
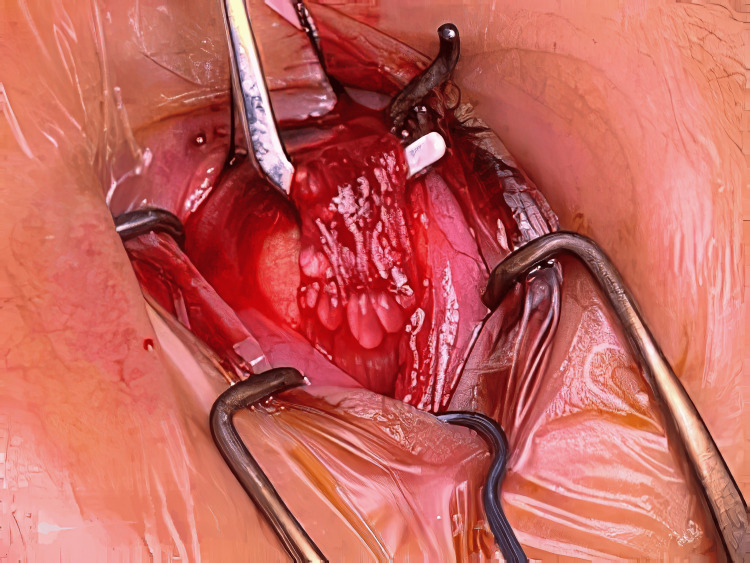
Intraoperative clinical photograph showing multiple cysts embedded on the lateral rectus muscle

**Figure 2:**
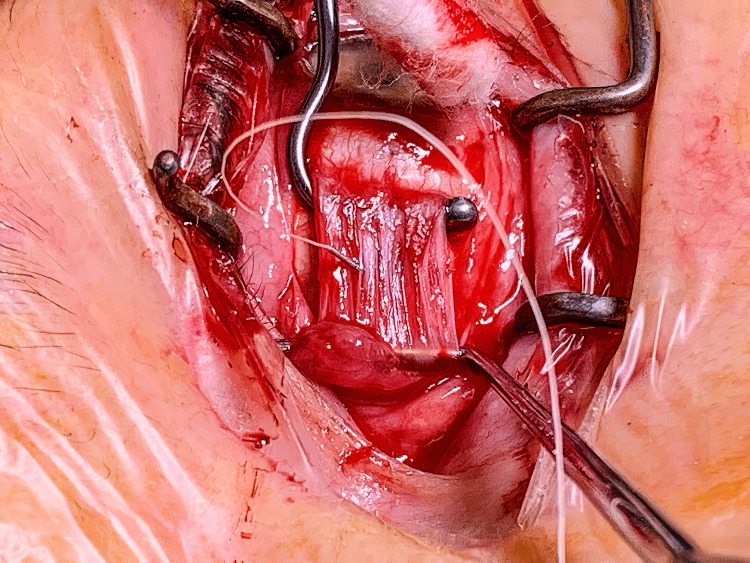
Intraoperative clinical photograph of the same lateral muscle following cyst removal

A lateral rectus recession of 5 mm and medial rectus resection of 4 mm was performed in February 2022. During the surgery, multiple cysts were found attached to the substance of the lateral rectus muscle, as shown in Figure 1. No cysts were noted on the medial rectus muscle. The cysts were removed either by whole excision or rupture. Figure 2 shows the complete removal of the cysts from the same lateral rectus muscle. The excised cysts were sent to pathology for a thorough histopathological examination. This confirmed sections of fat necrosis and lateral rectus muscle incorporating silicone oil. No other foreign material or sinister signs were confirmed. Postoperative examination revealed a satisfactorily corrected exotropia with an excellent cosmetic result.

## Discussion

Extraocular migration of intraocular silicone oil is a rare phenomenon. Numerous reports describe the migration of intraocular silicone oil to the subconjunctival space, orbit, eyelid, and central nervous system [1-9]. In this case, silicone oil cysts were discovered within the substance of the lateral rectus muscle, with the sparing of the other extraocular muscles.

It is challenging to ascertain the exact mechanism of this migration. However, various reasons for this can be postulated. One of the leading theories is that intraocular silicone oil may leak through pars plana vitrectomy ports during or following posterior segment surgery [10]. This is thought to be aggravated by postoperative spikes in intraocular pressure. Our patient underwent five vitreoretinal interventions in this case due to a complex retinal detachment and subsequent re-detachment.

Furthermore, silicone oil was left in situ long term due to the development of significant hypotony on removing the silicone oil. The combination of these factors may explain the silicone oil migration to the lateral rectus muscle. Migration of silicone oil to ocular structures can cause difficulties during strabismus surgery as the oil may become firmly adherent to nearby structures, thus preventing easy manipulation of extraocular muscles [3]. Although there was some difficulty in the excision of the oil cysts from the muscle tissue, we successfully corrected the strabismus and achieved a satisfactory cosmetic outcome.

In addition, it has been reported that intraocular silicone oil has an association with several complications, such as the development of cataracts, glaucoma, and epiretinal and subretinal fibrosis [10] and migration of silicone oil to extraocular muscle, as described in this report. Its emulsification and dispersion properties can explain silicone oil migration to numerous ocular structures [11]. In this case, our patient developed significant secondary glaucoma, ultimately controlled with beta-blocker/prostaglandin analog combination eye drops.

## Conclusions

Silicone oil introduced during vitreoretinal procedures as endotamponade may migrate to nearby ocular structures, including extraocular muscle, presenting as cysts within the muscle. This complication can cause difficulties during strabismus surgery. Therefore, surgeons should be alerted to this complication, particularly when planning to perform strabismus surgery in eyes with multiple previous vitreoretinal interventions utilizing silicone oil.
